# Baltimore Almshouse Infirmary

**Published:** 1830-07

**Authors:** 


### BALTIMORE ALMSHOUSE INFIRMARY.

### *Conscrescibility of the Blood.* By Dr. Wright.*

A young woman, name unknown, aged about twenty, full muscular form, and very fat, was brought to the Almshouse in a state of insensibility, on the 20th of March, 1829. No information could be obtained of the person who brought the woman, as to the cause or duration of her present state. He had been employed to take her from a house of ill fame in the city, and knew nothing more about her.

From the symptoms, and all that could be learned, the woman's condition was presumed to be the effect of intemperance, her insensibility the stupor of profound intoxication. She died almost immediately on being taken into the ward.

*Examination three or four hours after death.* Great sanguineous engorgement of all the tunics of the encephalon; both vascular

* American Journal of Med. Sciences.

systems fully injected; no special lesions by extravasation or palpable effusion. Stomach: Mucous coat generally florid around the pyloric orifice; all other viscera of the abdomen natural. Thorax: Lungs sound; gorged with blood, dark-coloured, and heavy. Direct cause of death uncertain, whether from congestive apoplexy, or fatal stupefaction by narcosis.

The subject of this case was brought to the institution in the interval between my visits, died directly after admission, and was examined by three highly intelligent students of the house, Dr. Smith, Dr. Warner, and Mr. Glassell. These gentlemen did me the favor to show me the heart of the subject, together with a body representing an exact cast of its chambers, of the canal of the aorta for five or six inches, (to where it was divided by themselves,) and of the tubes of the carotids and subclavians. This was a homogeneous coagulum, giving the figure of the cavities of the heart, and prolonged into solid cylinders, (five to six inches,) corresponding to the interior of the aorta, carotids, and subclavians; the cast of all the parts was of full natural size, the substance dense, and somewhat elastic. In the heart the concrete filled all the space completely, dipping between all the columnæ corneæ of the ventricles, and taking the impression of the musculi pectinati. The whole body was white and polished on the surface, and lively yellow-green within; a thin stratum of fluid red blood stained the surface of the coagulum in places. The gentlemen who made the examination represented that the trunk of both cavas was occupied by the same concrete matter, with a little fluid blood around the plug. Cylinders of the same matter were found in all the arterial and venous tubes as far as investigated, chiefly of the neck. The examination had not been carried to the principal trunks remote from the heart.

Does this process of concretion in the chambers of the heart, or the trunks of its great arteries, ever anticipate death? A man under treatment in one of our wards for chronic dyspepsia, not of aggravated character, was attacked about the 10th of Sept. (past,) by a sense of weight and pain of the chest, difficult breathing, chills and flushing, hot skin, feverish pulse, general bad feeling, anxiety, and aversion to lie down. He was bled, cupped across the thorax, and took a saline purge with antimonial combination. Next day, no better, hot skin, quick, sharp pulse, rather smaller, heaviness, with some pain in the breast; breathing slow, but embarrassed, not full; posture erect, sitting up; very small cough. Cupped on the sides, antimonial powders with nitre and gum Arabic, also the neutral mixture. The symptoms aggravated in the evening, oppression and labour of breathing increased, extremities became cold, circulation ebbed rapidly, and he sunk in the course of the night.

*Examination.* Subacute inflammation of some central portion of the lung had been suspected in the case. We found the lungs pale, soft, and natural throughout, no surface or central inflammation, congestion, or condensation, some hydropic infiltration of both pulmo-pleural sacs. The pericardium was half filled with bloody water, the inner surface of the bag pink-red colour. The surface of the heart was dark red; the coronary veins large and congested, those of the base of the heart, the posterior coronaries, stood full above the surface of the heart, gorged with black blood, and three of them larger than goosequills. Handling the heart, or pressure on those veins, did not at all empty them. The left ventricle was empty, the semilunar valves at the root of the aorta were fibro-cartilaginous; a small bone in one of them. The right ventricle empty. The right auricle was completely filled by a grayish-white concrete, of much firmness, which, occupying the whole sinus, the fossa ovalis above, and the expanded portion of the lower cava, choked the entire cavity of the auricle. The coronary veins, as before said, were all turgid; both cavas, and all the veins of the neck and thorax, full (fluid blood) to distention.

In this case there was old, partial hydrothorax. The acute seizure seems to have been carditis, more mischievous, perhaps, from complication with degenerescence of the valves. When the concrete in the auricle was formed, or what its sum, or kind of interference in the case, I am unable to say. It is the first considerable formation of the kind I have ever seen in that chamber.

